# Supplementary material for: The Rnf complex is a Na+ coupled respiratory enzyme in a fermenting bacterium, Thermotoga maritima
Source: Commun Biol. 2020 Aug 7;3:431. doi: 10.1038/s42003-020-01158-y (PMC7414866; doi:10.1038/s42003-020-01158-y)
Supplement: Supplementary file 1 — Supplementary Information [file 42003_2020_1158_MOESM1_ESM.pdf]

Supplementary Figures

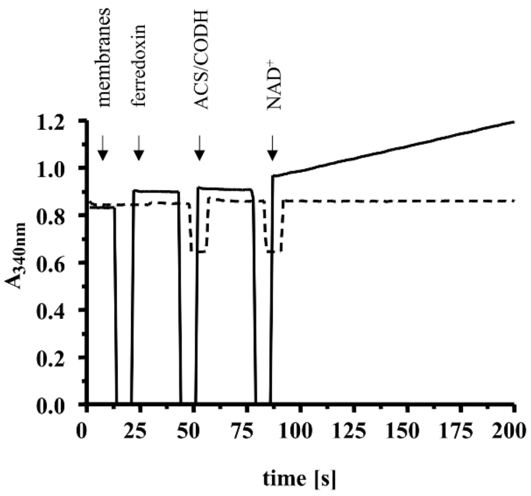

Supplementary Figure 1. Membranes of *T. maritima* catalyze Fd^{2-}:NAD$^+$-oxidoreductase activity. 100 µg membranes of *T. maritima* were added to 1 ml buffer (20 mM Tris-HCl, 20 mM NaCl 2 mM DTE, 2.2 µM resazurin, pH 7.7) and ferredoxin:NAD$^+$ oxidoreductase was measured as described in Material and Methods with (—) and without (---) addition of 30 µl NAD$^+$ (100 mM). The absorption was followed at 340 nm.

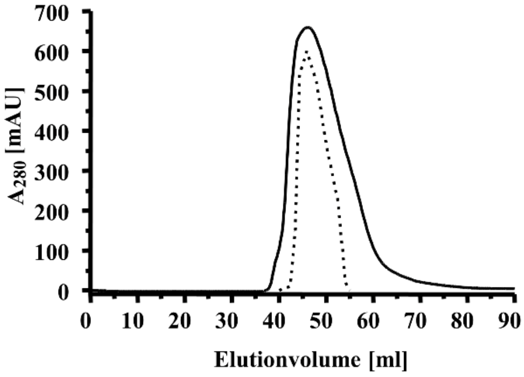

Supplementary Figure 2. Size exclusion chromatography of the Rnf/F_1F_O-ATP synthase complex from *T. maritima*. The Q-sepharose pool was applied on a Sephacryl S300 column equilibrated with buffer C (50 mM Tris-HCl, 20 mM $MgSO_4$, 150 mM NaCl, 2 mM DTE, 4.4 µM resazurin, 0.02 % DDM [w/v], 5 µM FMN, pH 8.0). The protein was separated with a flowrate of 0.5 ml/min and the separation was followed by measuring the extinction at 280 nm (▬). After 46 ml the main protein eluted from the column in one peak. The dotted line (----) indicates the ferredoxin:NAD^+ oxidoreductase activity. mAu, milli absorbance units.

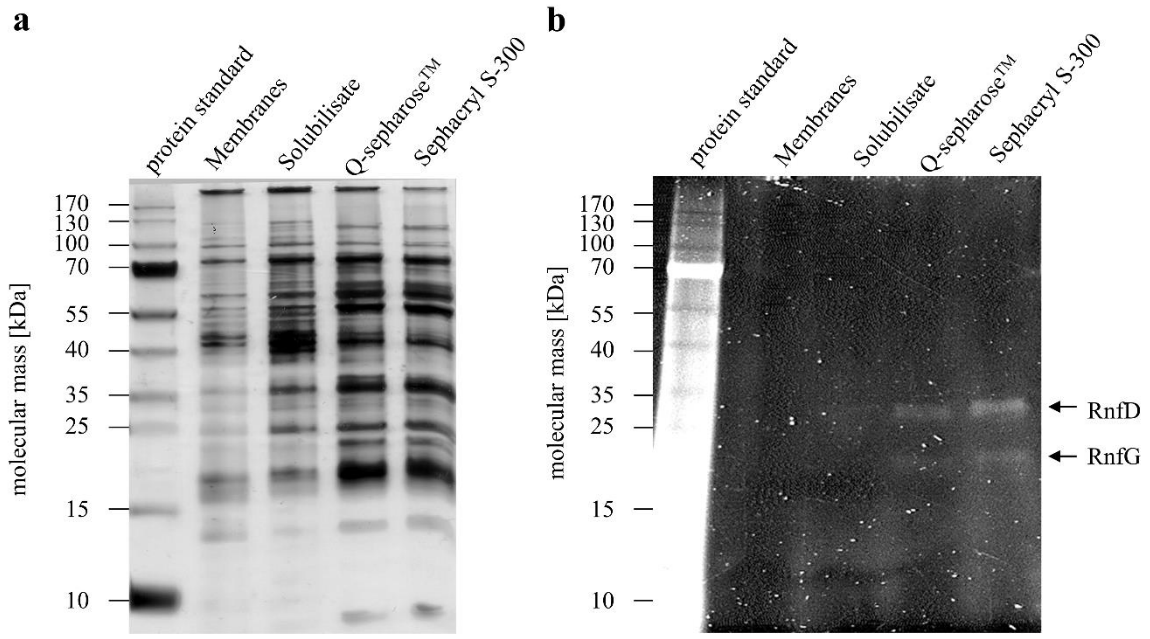

Supplementary Figure 3. Purification of Rnf/F_1F_O-ATP synthase complex from *T. maritima*. 20 µg protein from each purification step were separated on a 12.5% SDS-gel according to Laemmli *et al.* [1]. The proteins were visualized by staining with silver (a) or under UV light (b). The “Page Ruler Prestained Protein Ladder” (Thermo Fisher Scientific, Waltham, Massachusetts, USA) was used as protein standard.

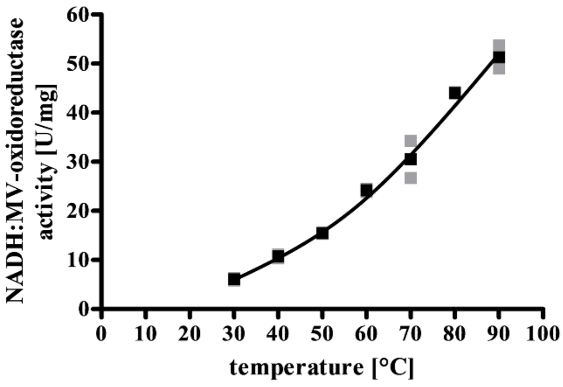

Supplementary Figure 4. Temperature dependence of the NADH:MV-oxidoreductase activity of the Rnf complex from *T. maritima*. 2.5 µg of the purified protein was added to 1 ml buffer (20 mM Tris-HCl, pH 7.7, 20 mM NaCl, 2 mM DTE, 4 µM Resazurin) and were incubated for 5 min at the corresponding temperature. The reaction was started by adding 10 µl MV (1 M) and 30 µl NADH (100 mM), and the absorption was followed by 604 nm. The data represent N = 2 independent experiments.

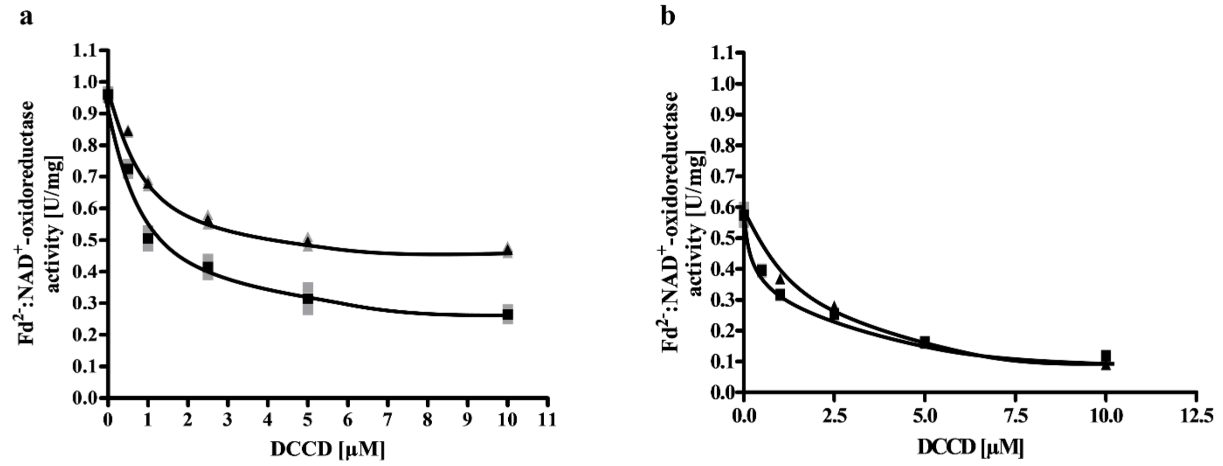

Supplementary Figure 5. Effect of Li^+ (A) and K^+ (B) on DCCD inhibition of ferredoxin:NAD^+ oxidoreductase activity. 14 µg of purified Rnf complex was added to 1 ml buffer (20 mM Tris-HCl, 2 mM DTE, 2.2 µM resazurin, pH 7.7) containing 0 - 10 µM DCCD in absence (■) or presence of 20 mM LiCl (A) or KCl (B) (▲). The sample was incubated for 30 min at room temperature followed by 5 min at 60 °C and ferredoxin:NAD^+ oxidoreductase was measured as described in Material and Methods. If LiCl or KCl was omitted during the preincubation, 20 mM LiCl (A) or KCl (B) was added before the reaction start. The data represent N = 2 independent experiments.

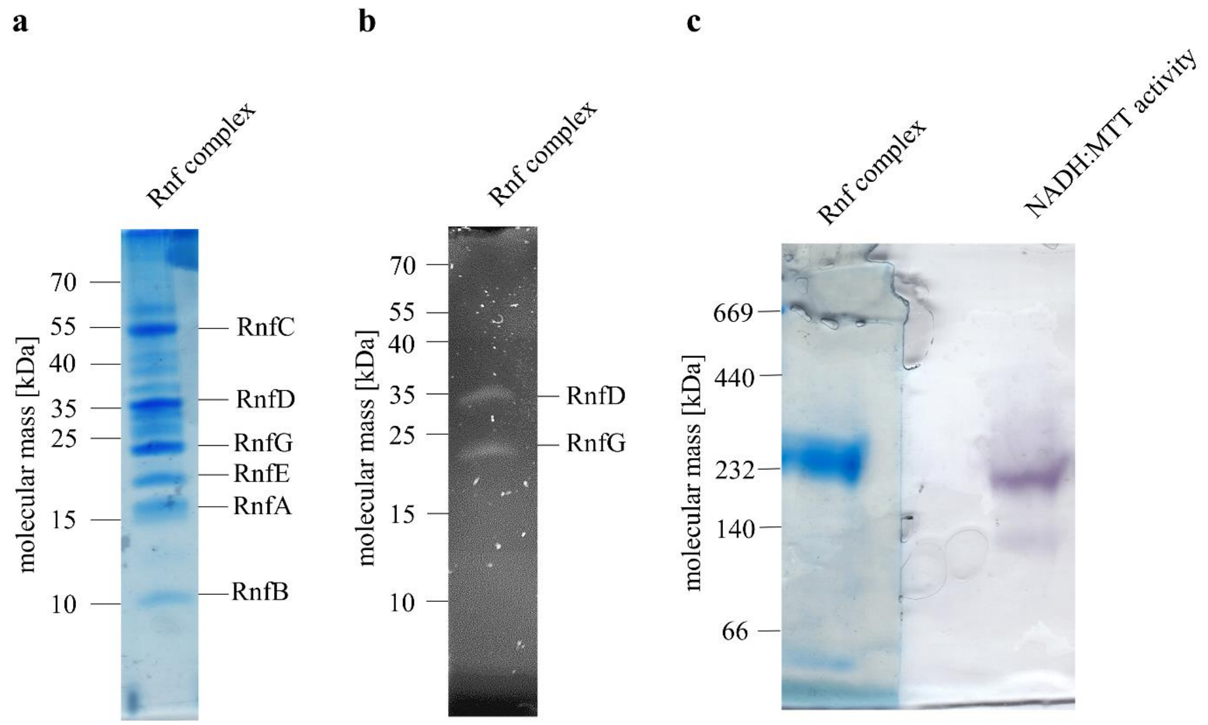

Supplementary Figure 6. Molecular mass of the purified Rnf complex and it´s subunit composition. 15 µg of the purified enzyme was either separated in a native PAGE (c) or under denaturating conditions in a SDS-PAGE (a). The gels were stained with Instant Blue™ (Expedeon, Cambridgeshire, UK) or with a NADH:MTT in gel activity assay under native conditions. The covalently bound FMN was visualized under UV-light (b).

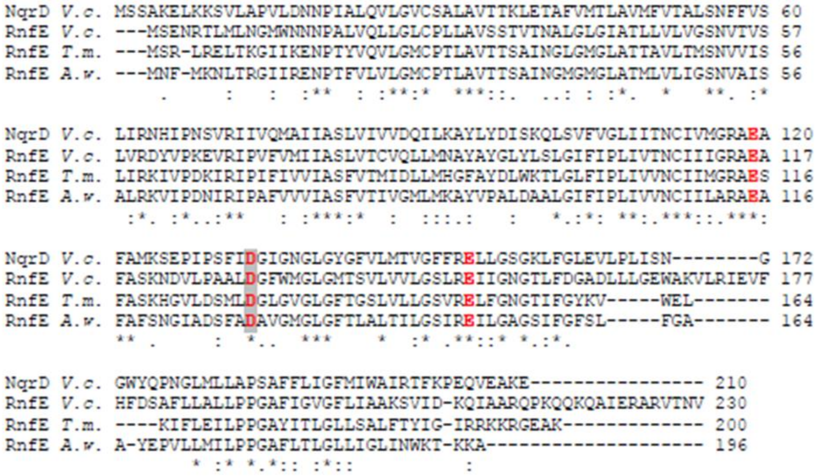

Supplementary Figure 7. Sequence alignment of NqrD from *V. cholerae* (*V. c.*) with RnfE from *A. woodii* (*A. w.*), *T. maritima* (*T. m.*) and *V. cholerae*. The conserved acidic amino acids are marked in red and the amino acid that is potentially involved in Na^+ binding is highlighted in gray. Positions with a star (*) are fully conserved residues. Positions with a colon (:) are residues with high similarities (reference value > 0.5 in Gonnet PAM 250 Matrix), dots (.) are residues with low similarities (reference value < 0.5 in Gonnet PAM 250 Matrix).

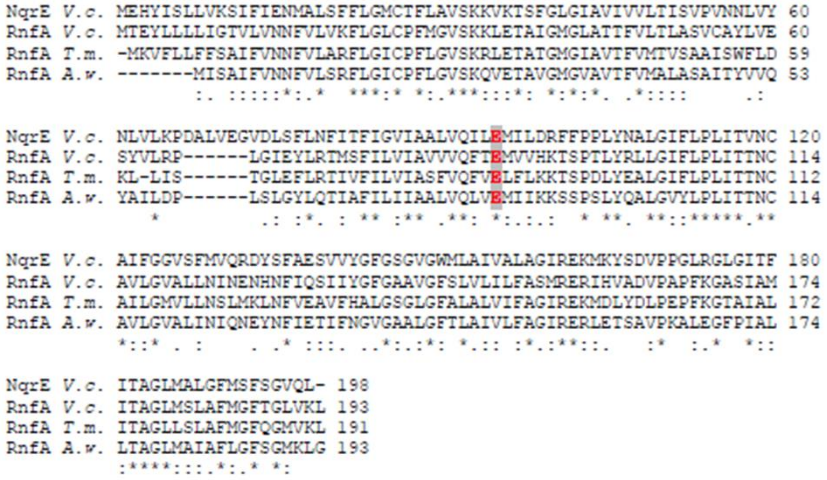

Supplementary Figure 8. Sequence alignment of NqrE from *V. cholerae* (*V. c.*) with RnfA from *A. woodii* (*A. w.*), *T. maritima* (*T. m.*) and *V. cholerae*. The conserved acidic amino acids are marked in red and the amino acid that is potentially involved in Na^+ binding is highlighted in gray. Positions with a star (*) are fully conserved residues. Positions with a colon (:) are residues with high similarities (reference value > 0.5 in Gonnet PAM 250 Matrix), dots (.) are residues with low similarities (reference value < 0.5 in Gonnet PAM 250 Matrix).

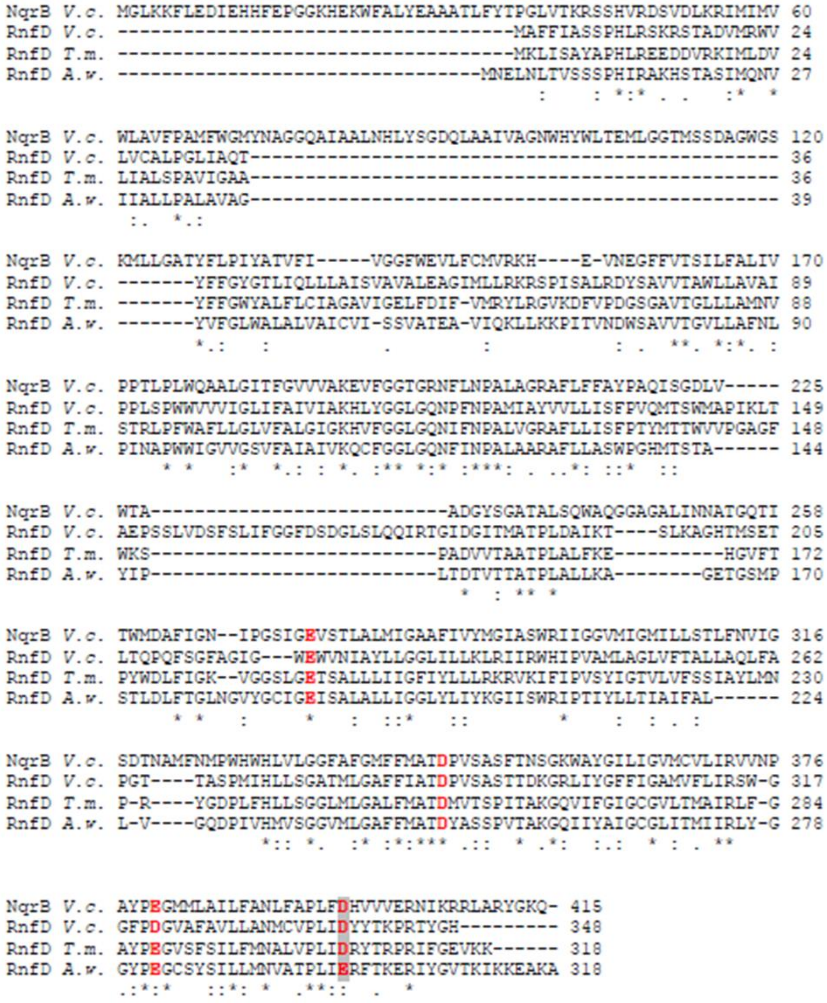

Supplementary Figure 9. Sequence alignment of NqrB from *V. cholerae* (*V. c.*) with RnfD from *A. woodii* (*A. w.*), *T. maritima* (*T. m.*) and *V. cholerae*. The conserved acidic amino acids are marked in red and the amino acid that is potentially involved in Na^+ binding is highlighted in gray. Positions with a star (*) are fully conserved residues. Positions with a colon (:) are residues with high similarities (reference value > 0.5 in Gonnet PAM 250 Matrix), dots (.) are residues with low similarities (reference value < 0.5 in Gonnet PAM 250 Matrix).

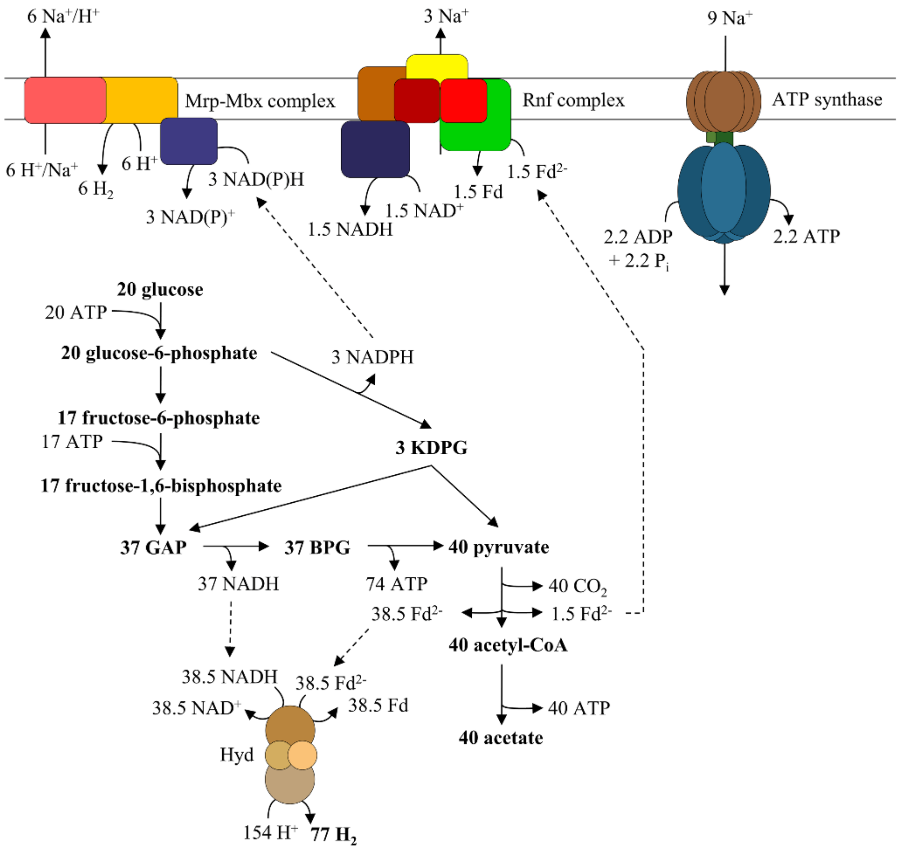

Supplementary Figure 10. A model of glucose metabolism linked to a Mrp-Mbx and Rnf complex in *T. maritima*. Glucose is oxidized *via* glycolysis and Entner-Doudoroff pathway and acetate is produced. NADPH generated in the gluose-6-phosphate dehydrogenase reaction is oxidized by a Mrp-Mbx complex by producing H_2, thereby establishing a Na^+ or H^+ gradient[2]. Electrons from Fd^{2-} are shuffled to NAD by the Rnf complex, thereby generating an additional Na^+ gradient; the Na^+ gradient is used by the F_1F_O ATP synthase to generate ATP. A potential H^+ gradient could be converted to a secondary Na^+ gradient *via* a Na^+/H^+ antiporter and then used for ATP synthesis or the ATP synthase may use Na^+ and H^+ simutaneously[3]. All other reducing equivalents are used by the electron bifurcating/confurcating hydrogenase (Hyd) to generate H_2. KDPG, 2-keto-3-desoxy-6-phosphogluconate, GAP, glyceraldehyde 3-phosphate, BPG, bisphosphoglycerate. Please note that the ion/electron and ion/ATP stoichiometries are based on thermodynamics (see also[4]).

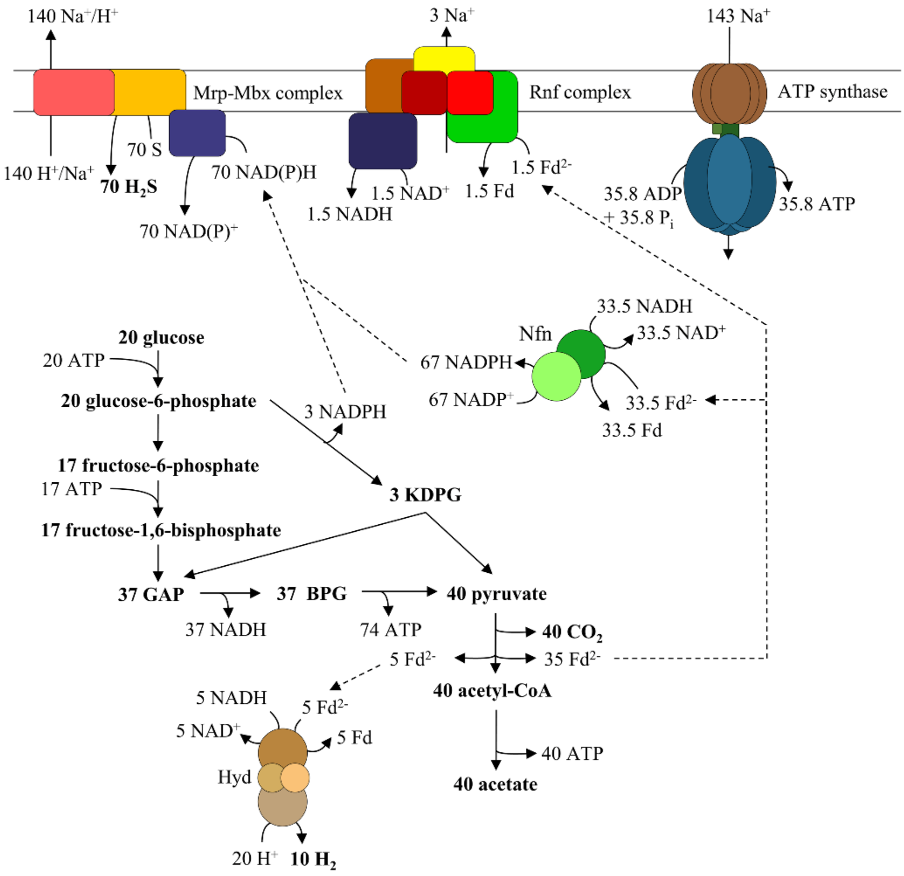

Supplementary Figure 11. A model of glucose metabolism linked to a Mrp-Mbx and Rnf complex in *T. maritima* under high H_2 partial pressure and presence of sulfur. Glucose is oxidized *via* glycolysis and Entner-Doudoroff pathway and acetate is produced. Most of the reducing equivalents are used from the NADH-dependent Fd^{2-}:$NADP^+$-transhydrogenase (Nfn) to confurcate electrons to $NADP^+$. NADPH generated in the gluose-6-phosphate dehydrogenase and transhydrogenase reaction is used by a Mrp-Mbx complex to reduce elemental sulfur to H_2S, thereby establishing a Na^+ or H^+ gradient[2]. A potential H^+ gradient could be converted to a secondary Na^+ gradient *via* a Na^+/H^+ antiporter and then used for ATP synthesis or the ATP synthase may use Na^+ and H^+ simultaneously[3]. Further electrons from Fd^{2-} are shuffled to NAD by the Rnf complex, thereby generating an additional Na^+ gradient; the Na^+ gradient is used by the F_1F_O ATP synthase to generate ATP. All remaining reduction equivalents are used by the electron bifurcating/ confurcating hydrogenase (Hyd) to generate H_2. KDPG, 2-keto-3-desoxy-6-phosphogluconate, GAP, glyceraldehyde 3-phosphate, BPG, bisphosphoglycerate. Please note that the ion/electron and ion/ATP stoichiometries are based on thermodynamics (see also[4]).

Supplementary Tables

Supplementary Table 1. Purification of the Rnf F_1F_O-ATP synthase complex from *T. maritima*.

Sample	Protein (mg)	Fd^{2-}:NAD^+-oxidoreductase activity (mU/mg)	total-activity (U)	enrichment	yield (%)
Membranes*	2200	97	213	1	100
Solubilisate	304	594	181	6.1	85
Q-Sepharose™	47	1340	63	13.8	30
Sephacryl S-300	13	2100	27	21.6	13

*Membranes were prepared from 30 g (wet weight) of cells

Supplementary Table 2. Proteins identified by LC-MS/MS and MALDI-TOF in the ATPase + Rnf complex preparation.

Protein	Accession number	Protein Idetification Propabillity	#Peptides	% Coverage
electron transporter RnfC [Thermotoga]	gi|490184336	100%	14	43%
electron transporter RnfG [Thermotoga]	gi|740209784	100%	8	31%
electron transporter RnfD [Thermotoga]	gi|740191358	32%	1	3.2%
ATP synthase epsilon chain [Thermotoga]	gi|490183443	100%	9	58%
ATP synthase subunit beta [Thermotoga]	gi|490183445	100%	27	60%
ATP synthase F1 subunit gamma [Thermotoga]	gi|490183448	100%	21	59%
ATP synthase subunit alpha [Thermotoga]	gi|490183450	100%	39	59%
ATP synthase subunit delta [Thermotoga]	gi|490183452	100%	13	54%
ATP synthase subunit B [Thermotoga]	gi|490183458	100%	19	71%
ATP synthase subunit c [Thermotoga]	gi|490183460	100%	3	32%
F0F1 ATP synthase subunit A [Thermotoga]	gi|490183462	100%	3	7.9%

Supplementary Table 3. Proteins identified by LC-MS/MS and MALDI-TOF in the Rnf complex preparation.

Protein	Accession number	Protein Idetification Propabillity	#Peptides	% Coverage
electron transport complex subunit RnfC [Thermotoga maritima]	G4FHF8_THEMA	100%	18	49%
electron transport complex subunit RnfG [Thermotoga maritima]	Q9WY88_THEMA	100%	12	44%
electron transport complex subunit RnfD [Thermotoga maritima]	Q9WY87_THEMA	100%	1	3.1%
electron transport complex protein RnfB [Thermotoga maritima]	G4FHG3_THEMA	100%	3	23%
electron transport complex subunit RnfA [Thermotoga maritima]	Q9WY90_THEMA	99%	1	5.8%

Supplementary References

1 Schägger, H. & von Jagow, G. Tricine-sodium dodecylsulfate-polyacrylamide gel electrophoresis for the separation of proteins in the range from 1 to 100 kDa. *Anal. Biochem.* **166**, 369-379 (1987).

2 Lim, J. K., Mayer, F., Kang, S. G. & Müller, V. Energy conservation by oxidation of formate to carbon dioxide and hydrogen via a sodium ion current in a hyperthermophilic archaeon. *Proc. Natl. Acad. Sci. U.S.A.* **111**, 11497-11502 (2014).

3 Schlegel, K., Leone, V., Faraldo-Gomez, J. D. & Müller, V. Promiscuous archaeal ATP synthase concurrently coupled to Na^+ and H^+ translocation. *Proc. Natl. Acad. Sci. U.S.A* **109**, 947-952 (2012).

4 Schut, G. J., Boyd, E. S., Peters, J. W. & Adams, M. W. The modular respiratory complexes involved in hydrogen and sulfur metabolism by heterotrophic hyperthermophilic archaea and their evolutionary implications. *FEMS Microbiol. Rev.* **37**, 182-203 (2013).
