# Supplementary material for: The Rnf complex is a Na+ coupled respiratory enzyme in a fermenting bacterium, Thermotoga maritima
Source: Commun Biol. 2020 Aug 7;3:431. doi: 10.1038/s42003-020-01158-y (PMC7414866; doi:10.1038/s42003-020-01158-y)
Supplement: Supplementary file 2 — Description of Additional Supplementary Files [file 42003_2020_1158_MOESM2_ESM.pdf]

Description of Additional Supplementary Files

File Name: **Supplementary Data 1**
Description: Raw datapoints for all figures from the main text and supplementary information.
